# Predicting Consumer Effort in Finding and Paying for Health Care: Expert Interviews and Claims Data Analysis

**DOI:** 10.2196/medinform.7892

**Published:** 2017-10-12

**Authors:** Sandra Long, Karen A Monsen, David Pieczkiewicz, Julian Wolfson, Saif Khairat

**Affiliations:** ^1^ University of Minnesota Minneapolis, MN United States; ^2^ University of North Carolina-Chapel Hill Chapel Hill, NC United States

**Keywords:** consumer health information, user effort, patient acceptance of health care, health expenditures, health services accessibility

## Abstract

**Background:**

For consumers to accept and use a health care information system, it must be easy to use, and the consumer must perceive it as being free from effort. Finding health care providers and paying for care are tasks that must be done to access treatment. These tasks require effort on the part of the consumer and can be frustrating when the goal of the consumer is primarily to receive treatments for better health.

**Objective:**

The aim of this study was to determine the factors that result in consumer effort when finding accessible health care. Having an understanding of these factors will help define requirements when designing health information systems.

**Methods:**

A panel of 12 subject matter experts was consulted and the data from 60 million medical claims were used to determine the factors contributing to effort.

**Results:**

Approximately 60 million claims were processed by the health care insurance organization in a 12-month duration with the population defined. Over 292 million diagnoses from claims were used to validate the panel input. The results of the study showed that the number of people in the consumer’s household, number of visits to providers outside the consumer’s insurance network, number of adjusted and denied medical claims, and number of consumer inquiries are a proxy for the level of effort in finding and paying for care. The effort level, so measured and weighted per expert panel recommendations, differed by diagnosis.

**Conclusions:**

This study provides an understanding of how consumers must put forth effort when engaging with a health care system to access care. For higher satisfaction and acceptance results, health care payers ideally will design and develop systems that facilitate an understanding of how to avoid denied claims, educate on the payment of claims to avoid adjustments, and quickly find providers of affordable care.

## Introduction

### Background

Technology is used by over 70% of the US population to seek health information [1,2]. For consumers to successfully improve health outcomes, they must be engaged and satisfied that the information they receive is accurate in meeting their needs [3,4]. This includes engagement in finding health care providers, deciding on appropriate treatments, and paying for care [3,5,6]. Therefore, to be fully engaged, the consumer must satisfactorily accept the design of the system or process they follow to access information [7].

When reviewing the literature related to the acceptance and engagement of health information systems, some of the most frequently occurring barriers include the failure of the system to meet consumer needs [3,8]. As the solutions created using proven methodologies are not fully meeting the needs of the consumer, it is likely that not all requirements were correctly identified [9]. The literature reviewed did not address the relationship of finding affordable health care to engagement with health care systems or how the monetary cost of to the consumer can have an impact on acceptance. By engaging with a well-designed health care system early on and planning their care, consumers can prevent the need to resolve ongoing payment or access issues that arise.

Usability acceptance and design methodologies have been created to make sure information delivery systems meet user requirements [7]. Some of these include human-centered design, Agile, Design for Six Sigma, and technology acceptance model [7,10-12]. All of these involve steps where the consumer requirements are documented and solutions are then created to meet them. These requirements ideally include aspects that make sure the consumer accepts using the system. There are two primary aspects that can be considered to lead to acceptance. The first is how much the consumer perceives the system to be useful or “the degree to which a person believes that using a particular system will enhance his or her performance or outcome” [13]. This means that in health care, the consumer trusts that the information they are receiving will lead to better health by using the system. The other is the perceived ease of use, or “the degree to which a person believes that using a particular system would be free from effort” [13]. This means that the least amount of effort required for the consumer to use the system leads to the most accepted design [7,13]. In this paper, effort is defined as the work done on the part of the consumer to find and pay for health care services. Health insurance payers and providers ideally consider this level of effort when developing information systems.

### Purpose and Aims

The purpose of this study was to explore how to design an accepted information system that assists consumers in accessing health care based on their diagnosis and the ability to easily find care. It focused on the ease-of-use or lack-of-effort aspect of acceptance. We aimed to define the types of users who put forth the most effort in accessing health care, resulting in improved consumer requirements for designing the health care system. The health care system of focus to this study is a call center that individuals, referred to as consumers, use to find a provider, understand payment for procedures, and get assistance with treatment decision support. It provides service to approximately 8 million consumers and receives 350,000 contacts from consumers per month. The consumer’s health insurance provider gives the consumer a telephone number and secure electronic mail address that can be used to contact the call center. The system was created with a primary focus of operational efficiency and to quickly answer only the question the consumer is asking or to transfer the call to someone else who can answer. The person who answers the questions used desktop systems and databases to answer the questions. There are times when the consumer is not aware of additional aspects of health care access, so they may not ask all questions related to how finding care and payment may work. This can be frustrating when they must talk to several people, are later surprised to find out that a provider is not accepting patients, or that their care was not covered by their insurance plan after they received treatment. Few of those using the system actually utilize the treatment decision support and clinical aspects because they are frustrated with the amount of effort required with payment and access.

The treatment and amount of care required depends on a diagnosis. We hypothesized that consumers with certain diagnoses utilize the system more often for administrative issue resolution, regardless of the clinical complexity of the diagnosis. Therefore, in this study, we explored how diagnosis contributes to a consumer’s understanding of how to access treatment.

## Methods

### Procedure

This study utilized expert knowledge and descriptive statistics to understand which variables predict effort and how they relate to diagnosis. A panel of experts within the organization was consulted to define which factors show whether a consumer is putting forth effort when accessing care. The panel consisted of 12 subject matter experts; 3 administrative call agents, 3 registered nurses who work directly with consumers to find care and assist with treatment decision support, 3 medical claims adjustors, and 3 data analysts familiar with medical claim and contact center data. They were recommended as being experts by other employees within the organization because of their health care education and experience credentials and were recruited through a conversation to determine their availability in assisting with the study. These experts had over 70 years of total experience working in various health care organizations throughout the United States, such as large hospital systems, health payer organizations, and clinical data analytic firms. Interviews were conducted individually with each panel member and consisted of two primary questions. The panel was first asked to define activities that contribute to a consumer’s effort in paying for health care. Second, the panel was asked to state what defines effort when trying to find providers and treatments. When the majority of the panel’s qualitative responses identified the same type of activity, that activity was determined to be an important factor. Validation of these factors also occurred through qualitative comparison with freeform responses from over 1000 consumers surveyed after their use of the system. Once the panel defined the factors, they were instructed to consider how the factors compare with each other and weigh the importance of the factors, giving higher weightage to those causing more effort.

Given that it is hypothesized that these factors occur more frequently for certain diagnoses, data needed to be analyzed to assign a value for each factor to each diagnosis. By giving a numerical value for each factor, it can be understood which diagnoses require more effort than others. The data related to payment and accessing care can be found in historical medical claims and records of consumer interactions with the health care organization.

### Data Analysis

**Figure 1:**
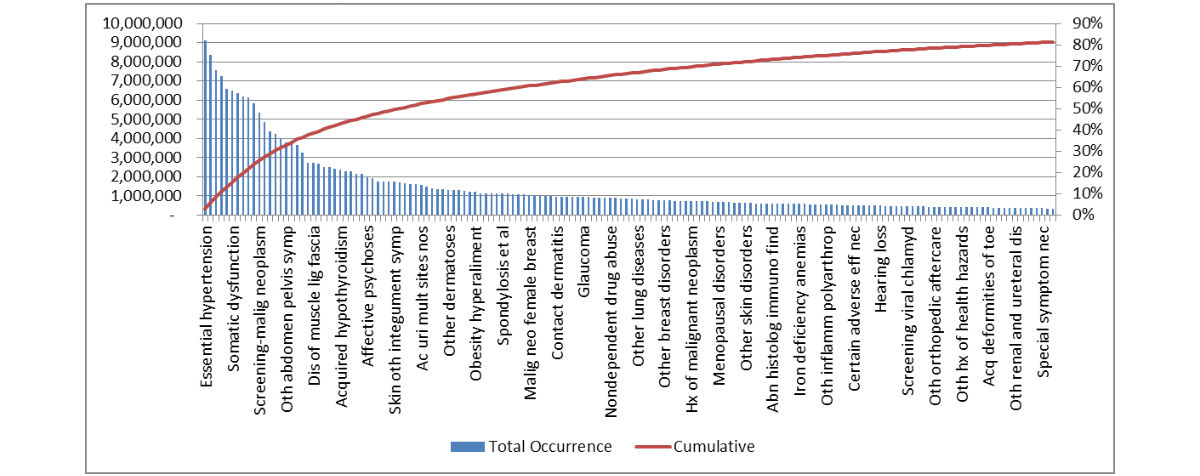
Diagnoses sorted by occurrence where left axis is the number of diagnoses occurring in the dataset and right axis is the percentage of the dataset made up by the diagnoses.

In this study, 12 months of health insurance claims data were used for analysis. It consisted of all data for the year of 2014, and all analysis was completed within the health care organization’s secure system to maintain privacy of personal information. The data included the name and address of providers, International Classification of Diseases (ICD) codes, payment processing information, and consumer identification information that could be matched to their demographics and utilization of the system being studied. To narrow the scope, the top 79.89% (233M/292M) of occurring diagnoses were used. This allowed for the majority of consumers to be considered in the study, but it did eliminate those consumers with only rare disease diagnoses. This was a negligible number of consumers as most with a rare diagnosis have a comorbidity diagnosis that was common. The justification for using 1 years’ worth of data was because this is the standard period for consumers to have the same health insurance plan. They typically select a new plan on a yearly basis, and even if they remain with the same one, the payer (government, employer, or administrator) often changes the benefits and coverage offered each year [14]. Provider association with the plan also changes. These yearly changes mean that the consumer must seek how to access and pay for care on a yearly basis, even if they are undergoing treatments similar to the prior year [14]. Although it is true that the treatment needed by a diagnosis may span more than 1 calendar year, the large quantity of data available eliminated concerns related to consumers being diagnosed at different points through the year. When grouping the diagnoses by ICD code, 117 codes made up 80% of all diagnoses occurring. Figure 1 shows the diagnoses used in the study and the volume of their overall contribution to the population. The mean and median were then calculated for the number of times a consumer experienced each of the effort factors defined by the expert panel.

## Results

Approximately 60 million claims were processed by the health care insurance organization in a 12-month duration with the population defined. About 292 million diagnoses appeared on the claims. When looking at the effort required to pay for care, the expert panel agreed that the primary factors are the number of denied claims, number of claims adjustments (when a claim was processed incorrectly the first time and then needed to be reworked), and when a consumer visits a medical provider who is out of network for their insurance coverage. Their reasoning for this was that a denied claim means that the burden of payment is then placed on the consumer who may have expected their insurance plan to cover the full or part of the cost. It is usually the consumer who must notice and initiate action for correction when the medical claims are processed incorrectly and need adjustment. Adjustments result in delayed payments as the medical claims are being reworked, causing the provider to bill the consumer until payment is received. Visits to network providers who are out of their insurance coverage result in high cost to consumers because the costs of care are not negotiated between the provider and the payer.

When the expert panel was asked what defines effort as far as accessing care, frequency of phone calls to the health care organization, number of Web portal visits, and number of mobile app usages were determined to be the main factors. These instances show that a consumer was not sure on how to access care, so they needed to contact the health care organization with inquiries. The types of inquiries from consumers consist of finding providers, determining the best treatments, and understanding the different types of care available within their health insurance plans. Other questions related to how to use their health insurance plan to pay for care also comes in through these communication channels, which ties to the effort required in covering the cost of treatment.

During the questioning, the experts also repeatedly brought up that the amount of effort often depends on the number of people with health care claims in a family or household. Their reasoning for this was that multiple members in the household may need care, and the majority of the administrative burden falls to a single person acting as the caretaker. Due to this, the number of people needing care in a household was also included as a factor. This also helped to define a consumer as all the members in a household or all the members needing care on a single health insurance plan subscribed to by the caretaker. The factors contributing to effort and weighting of importance assigned by the expert panel can be found in Table 1. The total effort put forth is considered to be 100%. The weightings were assigned based on how much each factor contributes to the total effort put forth by the consumer. Therefore, if half of the total effort came from a given factor, the weighting assigned would be 50%. The information provided by the panel aligned to the qualitative feedback consumers gave in surveys after using the system. The consumers were often frustrated when they received a bill from a provider for an unexpected amount of money and when they needed to contact the organization many times for resolution.

**Table 1:** Factors determined to represent consumer effort in accessing care and weighting of importance; summing the contribution of the factors equals total effort.

Factor of consumer effort	Weight of contribution to total (%)
Number of calls made by consumer	40
Number of claims where payment was denied by payer	25
Number of Web or mobile app visits	15
Visits to providers outside health insurance network	8
Number of adjustments required on claims	7
Number of people with claims in a household	5

Using existing data in the organization, the average number of times a consumer experienced each of the factors was determined. Therefore, each diagnosis had a value assigned for the average number of denied claims per consumer, average out-of-network usage per consumer, average number of claim adjustments per consumer, and average inquiries into the health care organization per consumer (phone, Web, or mobile app). The average number of people in a household was also calculated per consumer diagnosis.

The average of each effort factor was compared with the diagnosis. When the diagnoses were sorted based on the average of each individual factor, the list of those showing up at the top varied. For example, chronic kidney disease was the diagnosis with the second highest out-of-network provider visits per consumer but fell about halfway down the list when looking at how frequently the average consumer calls the health care organization. Correlations between factors were calculated to see how finding care and paying for it may relate. Although some variables are moderately correlated, each one is independent as it relates to the type of effort required. They are clinically significant as one action may lead to another. For instance, a consumer may have a denied claim, which may then qualify for an adjustment. Thus, they call or visit the portal to resolve the issue. The number of people in a household moderately correlates with all factors, with the exception of claim adjustments. An explanation for why there are not high correlations may be that one household member may have a very complex circumstance versus multiple household members having situations that are more easily resolved. The expert panel was consulted again to understand whether there is causation between variables. They concluded that although a person with a certain diagnosis may have trouble finding and paying for care, the effort required for one factor does not mean effort will be required for another factor. Given it is known that some factors require more effort than others based on the expert panel, the weightings defined by them were used to assign a score of effort to each diagnosis. This helped to define which diagnosis requires more effort than others.

The weightings were used similar to coefficients in a mathematical equation. The weighting of each factor was multiplied by the average number of occurrences for the factor. The results were then summed together to provide a total effort risk score for each diagnosis. See Table 2 for example of calculation.

**Table 2:** Calculation of effort risk score for dislocation of the knee.

Variable	Weight (%)	Average	Score
Number of people with claims in a household	5	2.14	0.11
Visits to providers outside health insurance network	8	4.48	0.36
Number of adjustments required on claims	7	0.57	0.04
Number of claims where payment was denied by payer	25	7.11	1.77
Number of calls made by consumer	40	0.65	0.26
Number of Web or mobile app visits	15	1.72	0.26
Total effort			2.80

Once all the scores were calculated, it was shown that the diagnoses with the highest amount of effort are not necessarily the ones that are most clinically complex. For example, both sprain of the knee and leg and malignant neoplasm of the breast show up in the top 20 of the list. The typical treatment within a given year for a sprained knee typically consists of less treatments in a year compared with breast cancer [15,16]. The final results are a list of the most frequently occurring diagnoses sorted, as they related to the effort a consumer must put forth with the health care system to find and pay for care. The list was given to the subject matter experts for validation. The top 25 diagnoses are shown in Table 3.

**Table 3:** Top 25 high-effort diagnoses.

Diagnosis description (ICD^a^ short description)	Average number of people in a household	Average number of out-of-network visits	Average number of claim adjustments	Average number of claim denials	Average number of phone inquiries	Average number of Web or mobile app inquiries	Total effort risk
Chronic kidney disease	1.61	7.10	1.38	15.87	0.57	1.13	5.11
Drug dependence	2.18	11.62	0.97	8.61	0.95	1.79	3.91
Heart failure	1.39	3.64	0.77	12.61	0.41	0.55	3.82
Complic medical care nec/nos	1.61	0.74	0.28	11.89	0.69	1.17	3.58
Sprain of the knee and leg	2.32	5.46	0.60	8.27	0.81	1.72	3.25
Chr airway obstruct nec	1.41	2.45	0.46	10.38	0.35	0.85	3.16
Malig neo female breast	1.74	3.40	0.97	7.89	0.85	2.47	3.11
Intervertebral disc dis	1.91	5.17	0.64	8.00	0.74	1.41	3.06
Somatic dysfunction	2.18	6.17	0.52	7.69	0.56	1.74	3.05
Dis of muscle or lig or fascia	2.00	5.33	0.47	7.49	0.63	1.48	2.91
Other cervical spice dis	1.96	5.04	0.52	7.14	0.68	1.68	2.85
Periph enthesopathies	1.95	4.27	0.52	7.22	0.67	1.78	2.82
Dislocation of the knee	2.14	4.48	0.57	7.11	0.65	1.72	2.80
Osteoarthrosis etal	1.59	3.33	0.66	7.20	0.74	1.64	2.73
Cardiac dysrhythmias	1.56	2.34	0.50	8.03	0.51	1.20	2.69
Nurit or metab or devel symp	2.40	3.97	0.72	6.28	0.75	1.53	2.59
Malign neopl prostrate	1.69	2.52	0.71	7.04	0.60	1.56	2.57
Back disorder nec and nos	1.87	4.04	0.47	6.23	0.60	1.54	2.48
Radius and ulna fracture	2.51	3.41	0.61	5.98	0.71	1.56	2.45
Oth-ill def morbid or mortl	1.55	2.15	0.33	6.80	0.62	1.41	2.43
Joint disorder nec and nos	1.96	3.64	0.46	5.88	0.69	1.42	2.38
Oth dis synov or tend or bursa	1.82	3.37	0.52	5.88	0.61	1.55	2.34
Normal pregnancy	2.19	2.45	0.59	5.16	1.06	1.84	2.34
Oth chr ischemic hrt dis	1.56	1.97	0.53	6.43	0.48	1.26	2.26
Sprain of the back nec or nos	1.84	4.02	0.37	5.39	0.54	1.10	2.17

^a^ICD: International Classification of Diseases.

## Discussion

### Principal Findings

This study took into consideration the consumer’s ability to pay and access health care with regard to their diagnosis. It was determined that having denied medical claims and inability to work with a provider covered by insurance results in higher effort for the consumer. Confusion and inquiries on how to get care also contribute to the need to engage. Removing the amount of work involved with these administrative tasks allows for easier access to the treatment procedures that are more likely to result in better health. Effort in using the health care system impacts a consumer’s willingness to accept the system and engage. This amount of effort also ties to diagnosis.

Diagnosis appears to stand out as being a way to determine who puts forth effort when accessing the health care system. Segmenting the population by diagnosis as it relates to effort will allow more customization to consumer needs when designing the system. By focusing the system design to assist those who put forth the most effort when accessing and paying for care, the overall satisfaction and acceptance of the system will be improved. Given that claim denials are the greatest source of effort, the system could help educate consumers on how to avoid this situation. The cost of care can be reduced through improved utilization of the broader health care system, and the cost of the information system within the health insurance organization should also be reduced as there will be less contacts and rework, thereby resulting in better operational efficiency.

Future studies could verify the belief that designing specifically for effort factors and diagnosis will improve the satisfaction of the consumer in using the health care system. In this study, payment and access systems were the area of focus, and health care consisted of other tasks such as treatment regimens, medication adherence, and clinical-based care. These additional tasks could also add to the level of effort; removing concerns about access and payment will only begin to make overall health care easier for consumers.

The method used in the paper can be replicated in other organizations to assist with guiding consumers toward accessible health care. Although this study included hundreds of variations of health insurance policies, call center representative expertise, provider networks, and population demographics across the United States, the results are likely dependent on the context of these factors in the organization studied. Ideally, a health care organization would take into consideration the structure and processes of their own system to determine the factors that result in effort for their consumers.

### Limitations

Limitations of this study include the fact that only 1 years’ worth of data were used. It is possible that over a lifetime, consumers with chronic diagnoses put forth more effort. Although this is true, consumers who do not typically use the health care system are often those most confused about how to use it [14]. Many of the highest effort diagnoses were related to injuries such as sprains and fractures, as well as normal pregnancy. Those who are athletic and mothers who are generally healthy would be those likely to experience these diagnoses and therefore would not be frequent users of the health care system.

### Conclusions

This study provides an understanding of how consumers must put forth effort when engaging with a health care system to access care. It shows how their diagnosis relates to the amount of effort put forth in administrative tasks such as finding providers and paying for care versus the effort related to undergoing treatments. It is known that for consumers to accept and engage with a system, it must be free from effort and easy to use. Therefore, designing systems using results found in this study is more likely to lead to better consumer engagement. For higher satisfaction and acceptance results, health care payers ideally will design and develop systems that facilitate an understanding of how to avoid denied claims, educate on the payment of claims to avoid adjustments, and quickly find providers of affordable care. This could be done across platforms that provide information for accessing care, such as forms, Web portals, and call centers. Consumers would receive information as part of the system process instead of relying on their own knowledge as a guide for health care navigation. There is a relationship between consumers’ ability to access and pay for care with their satisfaction in engagement; by first removing stress and improving satisfaction by finding financially accessible care, we can then gain consumer engagement for treatments and clinically related health and well-being.

## Abbreviations

ICD: International Classification of Diseases
